# Disability weights for the burden of oral disease in South Australia

**DOI:** 10.1186/1478-7954-2-7

**Published:** 2004-09-03

**Authors:** David S Brennan, A John Spencer

**Affiliations:** 1 Senior Research Fellow, AIHW Dental Statistics and Research Unit, Australian Research Centre for Population Oral Health, Dental School, Faculty of Health Sciences, The University of Adelaide, South Australia; 2 Professor, Social and Preventive Dentistry, AIHW Dental Statistics and Research Unit, Australian Research Centre for Population Oral Health, Dental School, Faculty of Health Sciences, The University of Adelaide, South Australia

**Keywords:** Oral disease, Burden of disease, EuroQol, Disability weight

## Abstract

**Background:**

Australian burden of disease estimates appeared inconsistent with the reported repetitive and ubiquitous nature of dental problems. The aims of the study were to measure the nature, severity and duration of symptoms for specific oral conditions, and calculate disability weights from these measures.

**Methods:**

Data were collected in 2001–02 from a random sample of South Australian dentists using mailed self-complete questionnaires. Dentists recorded the diagnosis of dental problems and provided patients with self-complete questionnaires to record the nature, severity and duration of symptoms using the EuroQol instrument. Data were available from 378 dentists (response rate = 60%).

**Results:**

Disability weights were highest for pulpal infection (0.069), caries (0.044) and dentinal sensitivity (0.040), followed by denture problems (0.026), periodontal disease (0.023), failed restorations (0.019), tooth fractures (0.014) and tooth wear (0.011). Aesthetic problems had a low disability weight (0.002), and both recall/maintenance care and oral hygiene had adjusted weights of zero.

**Conclusions:**

Disability weights for caries (0.044), periodontal disease (0.023) and denture problems (0.026) in this study were higher than comparable oral health conditions in the Australian Burden of Disease and Injury Study (0.005 for caries involving a filling and 0.014 for caries involving an extraction, 0.007 for periodontal disease, and 0.004 for edentulism). A range of common problems such as pulpal infection, failed restorations and tooth fracture that were not included in the Australian Burden of Disease and Injury Study had relatively high disability weights. The inclusion of a fuller range of oral health problems along with revised disability weights would result in oral health accounting for a larger amount of disability than originally estimated.

## Background

Although dental problems are widespread in number and impose a large burden on society in terms of lost production, pain and suffering, and health expenditure there is a tendency to underestimate their importance due to the generally non-fatal nature of most oral diseases and complacency arising from acknowledged improvements in oral health, such as trends toward lower caries levels among children and decreased edentulism in adults. Australians spend $2.6 billion on dental services, some 5.4% of recurrent health expenditure for 1998–99 [1]. While dental diseases are not usually life-threatening, the importance of delivering services needs to be considered in view of the repetitive and ubiquitous nature of dental problems which combine to create a large burden. For example, dental problems were ranked as the fourth most frequent illness condition, behind headache, hypertension and colds in a two week survey period [2], dental caries (decay) has been ranked as the highest diet-related disease in Australia in terms of both total costs and health care costs [3], and periodontal (gum) disease has been reported to be the fifth most prevalent health condition in Australia [4].

The disability-adjusted life year or DALY [5,6] provides a summary measure of population health that combines information on the impact of premature death and of disability and other non-fatal health outcomes. The Australian Burden of Disease and Injury Study used the DALY approach to assess the magnitude and impact of health problems in Australia [7]. This burden of disease methodology is designed to inform health policy in relation to the prevention and treatment of health problems. This provides a different picture to traditional approaches that take into account deaths, but not disability. However, the authors acknowledge that further work is required to refine and develop the data and methods.

Estimates of DALYs are limited by inadequate information on the distribution of severity of disease and the course of a disease. Due to limitations in the data many of the disease models are necessarily simple and approximate, with their precision reflecting the source and nature of the data underlying the model. Also, the lack of Australian disease weights may mean that they are not completely representative of Australian societal preferences [7]. Hence the estimates of YLD (Years Lost due to Disability) and DALYs (Disability-Adjusted Life Years) should be regarded as provisional and developmental.

The DALY estimates for oral health in the Australian Burden of Disease and Injury Study seemed inconsistently low with other reports of the high prevalence and incidence of oral health conditions such as dental caries and periodontal disease [4]. There are a number of specific problems associated with estimating oral health DALYs. There is a lack of recent national data on oral health. Just one national oral health survey (NOHSA) has been performed in Australia, which was conducted in 1987–88 [8] and hence is now out of date. Data from other sources [9] indicates that oral health status in Australia is changing, which makes it difficult to estimate disease models for caries and periodontal disease based on data from NOHSA. Sequelae need to be included in disease models, for example disease models should account for sequelae of caries such as pulpal/periapical infection. Oral health estimates need to include a fuller range of oral conditions such as cuspal fractures. Edentulism estimates were based on self-reported data, and may be under-estimates if edentulous persons are less likely to participate in population surveys of oral health. The disease models were based on assumptions regarding severity and duration of symptoms that may require quantitative confirmation and revision.

The broad aims of the project were to evaluate methods used to measure the burden of disease associated with oral conditions in Australia. The specific aims were to obtain measures of burden of disease related to specific oral conditions, measure these in terms of the nature, severity and duration of symptoms, and calculate disability weights from these measures.

## Methods

### Design

The study was conducted using a 2-stage sampling design whereby dentists were randomly sampled from the South Australian Dental Register, randomised into one of seven equal-sized study groups and sent a mailed self-complete dentist questionnaire along with up to five self-complete patient questionnaires depending on the study group. Dentists were provided with a practitioner logbook in which to record for the first 1 to 5 adult patients (depending on study group assignment of dentist) of a random clinical day the treatment they performed and diagnosis of the oral disease or condition treated. At the conclusion of treatment the practitioner passed on a survey kit to their sampled patient(s) containing a patient questionnaire, cover letter and explanation sheet. Sampled patients completing the patient questionnaire recording basic socio-demographic characteristics and data concerning the nature, severity and duration of their symptoms. The patient questionnaires were identified using the practitioner identification number allowing linkage between the practitioner logbook data and patient questionnaire data, but maintaining the anonymity of each patient to the investigators.

### Sampling and data collection

The emphasis of the project was to obtain precise estimates of the component measures of the burden of oral disease. These are typically expressed as percentages, such as the percentage of persons or percentage of time experiencing symptoms of a given degree of severity. Taking a parameter size of 10% as a reference estimate for any given measure, in order to achieve a level of precision of 20% or less relative standard error, a minimum target sample of 225 patients was required. This would provide an acceptable minimum level of precision for estimates as low as 10% in size, and better precision for any estimates larger than 10% in size.

Data were collected during 2001–2 with a primary approach letter sent initially to each dentist, followed a week later by the survey materials, with a reminder card two weeks later, and up to four follow-up mailings of survey materials to dentists who had not yet responded in order to ensure higher response rates [10].

### Data items

Dentists recorded the details of the dental conditions that patients had, and patients recorded their experience of those dental conditions. Diagnosis of dental conditions was collected from dentists using an open-ended question in the dentist questionnaire and coded using the coding scheme adopted in the Longitudinal Study of Dentists' Practice Activity [11]. Data on dental conditions in both the practitioner logbook and patient questionnaire were collected for the main dental condition that was currently being treated, another dental condition being treated besides the main condition, and for dental conditions that were not currently treated. In the patient questionnaire, patients were asked if the dental conditions had caused problems in each of six health state dimensions, the severity of the problem (prevalence and percent of time that problems were experienced in relation to each health state dimension) and duration of problems in each dimension. The six health state dimensions were: mobility (e.g, walking about), self-care (e.g, washing, dressing), usual activities (e.g., work, study, housework, family or leisure), pain/discomfort, anxiety/depression and cognition (e.g, memory, concentration, coherence, IQ). They were measured using the European Quality of Life indicator or EuroQol (EQ-5D+) instrument [12]. The EuroQol measures each of these six dimensions according to a 3-level response grading from 1 = no problems, 2 = some / moderate problems and 3 = extreme problems.

### Data analysis

Following descriptive analysis of response rates and characteristics of respondents, the distribution of dental conditions was examined for the 11 most common dental conditions. These dental conditions were then examined in terms of the nature, severity and duration of each condition. Disability weights were then calculated for each dental condition by using a health state valuation algorithm based on UK population data [13]. A patient could have more than one dental condition and hence have more than one disability weight. This initial disability weight was then adjusted by multiplying the coefficients from the health state valuation by the percentage of time affected by the problem. A final adjustment to the disability weights was performed by subtracting the intercept term from the health state valuation equation from the disability weight that had been adjusted by the percentage of time affected by the problem (see appendix 1: additional file 1 for details), as there was some conjecture as to how such an intercept term should be interpreted [13]. For each type of disability weight (i.e., unadjusted and adjusted) a dental condition-specific weight was calculated as the average of the weights for each patient that had reported having that specific dental condition. Results are reported adjusted for the survey design effect of clustering of patient observations within the primary sampling unit of dentists [14]. Disability weights were also calculated using the multiplicative EQ-5D+ regression model from the Australian Burden of Disease and Injury Study [7] as a form of cross-validation of the approach (see appendix 2: additional file 1 for details).

## Results

### Response

A total of 378 dentists responded to the survey (response rate = 60%). Response rates between study groups ranged from 49% to 70% and tended to be higher in study groups that required dentists to sample less patients, but the effect was not monotonic (Table 1). Data were available for 375 patients from the patient questionnaire, comprising a response rate of 72% of patients sampled, with response rates between study groups ranging from 69% to 92%.

**Table 1:** Response to the practitioner logbook and patient questionnaires.

		Practitioner logbook				Patient questionnaire		
			
				Patients recorded		Patients recorded		
						
	Patients sampled per dentist	Number of dentists responding	Response rate (%)	Number	Percent	Number	Percent	Response rate (%)
Pilot study	5	60	(65)	135	(17.9)	93	(24.8)	(69)
Main study (a)	0	61	(70)	237	(31.4)	-	(-)	(-)
Main study (b)	1	56	(62)	37	(4.9)	29	(7.7)	(78)
Main study (c)	2	54	(60)	49	(6.5)	45	(12.0)	(92)
Main study (d)	3	43	(49)	61	(8.1)	41	(10.9)	(67)
Main study (e)	4	50	(58)	118	(15.6)	84	(22.4)	(71)
Main study (f)	5	54	(57)	119	(15.7)	83	(22.1)	(70)
Total		378	(60)	756	(100.0)	375	(100.0)	(72)

### Characteristics of patients

The characteristics of patients are presented in Table 2 where data from private general practice [11] and Australian population estimates [15,16] are presented for comparison. The majority of patients were female (59.5%), born in Australia (75.5%), had dental insurance (64.8%) and had visited a dentist in the last 12 months (65.3%). The main reason for dental visiting was for other dental problems not involving relief of pain (46.7%), followed by check-ups (35.2%) and emergency visits involving relief of pain (18.1%).

**Table 2:** Characteristics of patients in the Burden of Oral Disease Study compared with private general practice and Australian population estimates.

	Burden of Oral Disease Study	Private General Practice ^(a)^	Australian Population
	%	%	%
Sex			
% Female	59.5	54.9	^(b) ^50.4
Place of birth			
% Australian	75.5	n.a.	^(b) ^76.4
Dental insurance status			
% Insured	64.8	47.8	^(c) ^34.8
Reason for dental visit			
Check-up	35.2	41.1	^(c) ^45.1
Emergency	18.1	28.6	n.a.
Other dental problem	46.7	30.8	n.a.
Time since last dental visit			
% visited in last 12 months	65.3	n.a.	^(c) ^61.3

(a): Longitudinal Study of Dentists' Practice Activity 1998–99

(b): Australian Bureau of Statistics 2002

(c): National Dental Telephone Interview Survey 1999

n.a. : denotes data not available

### Dental condition

**Figure 1:**
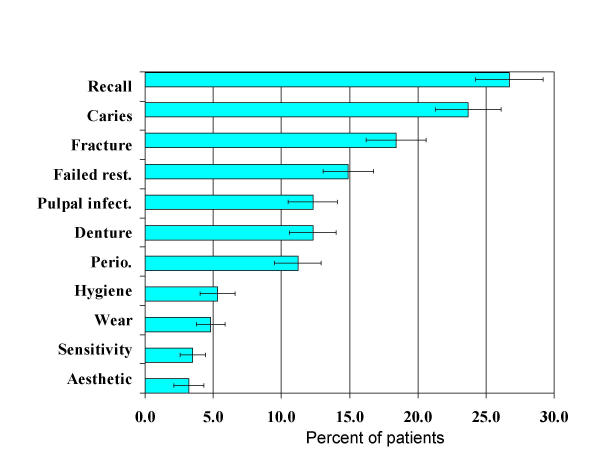
Distribution of dental conditions (% of patients ± SE).

The distribution of dental conditions is presented in Fig 1 for the 11 most common conditions. Recall/maintenance (26.7%) and caries (23.7%) were the most common conditions followed by tooth fracture (18.4%), failed restorations (14.9%), pulpal infection and denture problems (both 12.3%), and periodontal disease (11.2%). Further analysis assumes zero disability weights for the conditions of recall/maintenance care and oral hygiene conditions due to the lack of symptoms associated with them, and therefore excludes each of these conditions from further consideration.

### Dental conditions by health state dimensions and duration

Dental conditions are presented in Table 3 by health state dimensions and duration. A high percentage of patients reported problems (defined as level 2 = some/moderate or level 3 = extreme) with the dimension of pain or discomfort for problems such as pulpal infection (63%), dentinal sensitivity (55%), tooth wear (40%), caries and denture problems (both 38%) and tooth fracture and periodontal disease (both 35%). A high percentage of patients also reported problems with the dimension of anxiety or depression for problems such as periodontal disease (32%), tooth wear (30%) and dentinal sensitivity (27%). The percentage of time affected by dental conditions was generally high for most dimensions for dental conditions such as caries, tooth fracture, and denture problems, and for the dimensions of pain or discomfort and anxiety or depression for dental problems such as failed restoration, periodontal disease and pulpal infection. Aesthetics had the longest duration among dental conditions, however aesthetic problems comprised relatively low percentages of total conditions (as shown in Fig 1). Among the more common conditions caries and denture problems had long durations (ranging between 66 and 81 weeks).

**Table 3:** Distribution of health state dimensions (± SE) by dental conditions.

	Duration (weeks)		Health state dimensions					
			
	Mean		Mobility	Self-care	Usual activities	Pain / discomfort	Anxiety / depression	Cognition
Caries	81 ± 18	Prevalence ^(a)^	^(3)^4 ± 2	^(3)^3 ± 2	14 ± 4	38 ± 6	19 ± 5	^(1)^10 ± 3
		Time ^(b)^	^(3)^25 ± 14	33 ± 8	26 ± 6	42 ± 7	34 ± 9	29 ± 8
Fracture	^(1)^27 ± 9	Prevalence ^(a)^	^(3)^3 ± 2	^(3)^3 ± 3	^(3)^3 ± 2	35 ± 6	18 ± 5	^(2)^10 ± 4
		Time ^(b)^	†10	-	†50	28 ± 7	^(3)^20 ± 14	^(3)^55 ± 45
Denture problem	^(1)^66 ± 24	Prevalence ^(a)^	^(2)^13 ± 6	^(3)^3 ± 3	^(2)^16 ± 7	38 ± 10	^(1)^22 ± 7	^(1)^19 ± 7
		Time ^(b)^	^(3)^28 ± 24	†100	^(3)^40 ± 23	33 ± 6	^(1)^34 ± 10	^(3)^25 ± 14
Failed restoration	15 ± 4	Prevalence ^(a)^	0	0	^(3)^2 ± 2	27 ± 6	^(2)^10 ± 4	^(3)^4 ± 3
		Time ^(b)^	-	-	-	29 ± 8	^(2)^23 ± 11	^(3)^10 ± 10
Periodontal disease	^(1)^49 ± 19	Prevalence ^(a)^	^(3)^3 ± 3	0	^(3)^3 ± 3	35 ± 8	32 ± 9	^(3)^3 ± 3
		Time ^(b)^	-	-	-	32 ± 7	28 ± 7	-
Pulpal infection	31 ± 8	Prevalence ^(a)^	^(3)^2 ± 2	0	^(3)^12 ± 6	63 ± 8	^(1)^22 ± 7	^(1)^10 ± 5
		Time ^(b)^	†10	-	32 ± 9	46 ± 6	41 ± 6	^(1)^30 ± 12
Wear	^(1)^69 ± 23	Prevalence ^(a)^	0	0	0	^(2)^40 ± 16	^(3)^30 ± 15	^(3)^10 ± 10
		Time ^(b)^	-	-	-	^(2)^9 ± 4	^(3)^33 ± 17	†50
Sensitivity	^(2)^21 ± 9	Prevalence ^(a)^	0	0	0	55 ± 16	^(3)^27 ± 14	0
		Time ^(b)^	-	-	-	^(3)^21 ± 12	^(3)^28 ± 23	-
Aesthetics	^(2)^118 ± 51	Prevalence ^(a)^	0	0	0	^(3)^27 ± 19	^(3)^9 ± 9	^(3)^9 ± 9
		Time ^(b)^	-	-	-	^(3)^5 ± 3	†5	†25

(a) Percentage of patients reporting problems (at level 2 = some/moderate or level 3 = extreme) with a health state dimension related to the dental condition

(b) Percentage of time during the period that the patient had experienced reported symptoms or problems related to the dental problem

-: denotes no observations

†: denotes one observation only

(1): Relative standard error = 30–39%

(2): Relative standard error = 40–49%

(3): Relative standard error = 50–59%

### Dental conditions by disability weights

Unadjusted disability weights derived from the additive model (DW_a_) were highest for pulpal infection, dentinal sensitivity and caries, followed by denture problems, periodontal disease, tooth wear and tooth fractures (Table 4). When adjusted by the percentage of time that dental conditions were experienced all disability weights (DW_b_) were reduced. Pulpal infection remained the highest adjusted disability weight, followed by caries and dentinal sensitivity, followed by denture problems, periodontal disease and failed restorations. Subtracting the intercept from the unadjusted disability weight reduced all weights (DW_c_) by a constant amount.

**Table 4:** Disability weights (95% CI) by dental problem – derived from additive model.

	Unadjusted Disability Weight (DW_a_)	Disability Weight (DW_b_) adjusted by % time experienced problems	Disability Weight (DW_c_) adjusted by % time experienced problems minus intercept‡
Caries	0.185 (0.143–0.226)	0.125 (0.094–0.157)	0.044 (0.013–0.076)
Fracture	0.150 (0.123–0.178)	0.095 (0.085–0.105)	0.014 (0.004–0.024)
Denture problem	0.163 (0.124–0.200)	0.107 (0.084–0.130)	0.026 (0.003–0.049)
Failed restoration	0.136 (0.105–0.166)	0.100 (0.081–0.118)	0.019 (0.0001–0.037)
Periodontal disease	0.158 (0.123–0.194)	0.104 (0.090–0.119)	0.023 (0.009–0.038)
Pulpal infection	0.210 (0.162–0.258)	0.150 (0.110–0.191)	0.069 (0.029–0.110)
Wear	0.152 (0.093–0.210)	0.092 (0.078–0.107)	†0.011 (0.000–0.026)
Sensitivity	0.191 (0.102–0.281)	0.121 (0.045–0.198)	†0.040 (0.000–0.118)
Aesthetics	0.121 (0.067–0.175)	0.083 (0.080–0.086)	†0.002 (0.000–0.005)

† confidence interval truncated at zero

‡ standard error for DW_c _is the same as DW_b _due to transformation by a constant

The disability weights derived from the multiplicative model are presented in Table 5. The unadjusted disability weights derived from the multiplicative model (DW_d_) followed a similar rank order as for the unadjusted disability weights derived from the additive model (DW_a_), being highest for pulpal infection with caries ranked second-highest, but with some re-ordering of the next highest conditions (i.e., denture problems were ranked second rather than fourth, while dentinal sensitivity was ranked fourth rather than second, then followed in the same order by periodontal disease and tooth wear). When adjusted by the percent of time that dental conditions were experienced all disability weights derived from the multiplicative model (DW_e_) were reduced, with pulpal infection ranked highest, followed by caries, dentinal sensitivity, denture problems, tooth wear and periodontal disease.

**Table 5:** Disability weights (95% CI) by dental problem – derived from multiplicative model.

	Unadjusted Disability Weight (DW_d_)	Disability Weight (DW_e_) adjusted by % time experienced problems
Caries	0.121 (0.073–0.170)	0.059 (0.022–0.095)
Fracture	0.091 (0.050–0.132)	0.021 (0.006–0.035)
Denture problem	0.124 (0.070–0.179)	0.041 (0.007–0.075)
Failed restoration	0.065 (0.028–0.102)	0.030 (0.005–0.054)
Periodontal disease	0.106 (0.056–0.156)	0.034 (0.017–0.052)
Pulpal infection	0.128 (0.067–0.191)	0.076 (0.028–0.123)
Wear	0.099 (0.005–0.193)	†0.036 (0.000–0.083)
Sensitivity	0.112 (0.007–0.218)	†0.048 (0.000–0.138)
Aesthetics	†0.050 (0.000–0.110)	†0.002 (0.000–0.005)

† confidence interval truncated at zero

The final adjusted disability weights derived from the additive (DW_c_) and multiplicative (DW_e_) models were similar in rank ordering, with pulpal infection, caries, dentinal sensitivity and denture problems ranked highest. While the adjusted disability weights derived from both models were also similar in magnitude those derived from the additive model were lower for all oral conditions except aesthetics, which was identical for both models. In the remainder of the paper the final adjusted disability weights derived from the additive model (DW_c_) will be presented, as this provided the most conservative estimate.

### Comparison of disability weights

The disability weights for oral conditions are presented in Table 6 along with the weights for oral conditions from the Australian Burden of Disease and Injury Study [7]. Comparing edentulism with denture problems shows a higher disability weight in the Burden of Oral Disease Study estimate. For periodontal disease the disability weight estimate from the Burden of Oral Disease Study was higher. For caries, the disability weight was higher for the Burden of Oral Disease Study estimate than either of the two estimates for caries from the Australian Burden of Disease and Injury Study.

**Table 6:** Comparison of oral health disability weights by source.

Australian Burden of Disease and Injury Study		Burden of Oral Disease Study	
Edentulism		Denture problems	
Edentulism	0.004	Denture problem	0.026
Periodontal disease		Periodontal disease	
Periodontal disease	0.007	Periodontal disease	0.023
Caries		Caries	
Caries (filling)	0.005	Caries (all cases)	0.044
Caries (extraction)	0.014		

The disparity in disability weights for oral health conditions between the Australian Burden of Disease and Injury Study and the Burden of Oral Disease Study is examined further in Table 7, which compares the assumptions for oral health disability weights by source. Comparing edentulism estimates with those for denture problems shows a slightly higher estimate for percentage of time affected and a more marked difference in percentage of cases affected in the Burden of Oral Disease Study estimates. For periodontal disease the estimates from the Burden of Oral Disease Study are higher for both percentage of time and percentage of cases. For caries, estimates of percentage of time and duration for moderate pain and moderate anxiety were both higher for the Burden of Oral Disease Study, as was the estimate for extreme pain.

**Table 7:** Comparison of assumptions for oral health disability weights by source.

Australian Burden of Disease and Injury Study			Burden of Oral Disease Study^(a)^		
Edentulism	% of time	% of cases	Denture problems	%(± se) of time	%(± se) of cases
			
Moderate pain	25% of time	10% of cases	Moderate pain	33 ± 6% of time	38 ± 10% of cases
Moderate anxiety	25% of time	10% of cases	Moderate anxiety	^(1)^34 ± 10% of time	^(1)^22 ± 7% of cases
Periodontal disease	% of time	% of cases	Periodontal disease	%(± se) of time	%(± se) of cases
			
Moderate pain	10% of time	10% of cases	Moderate pain	30 ± 8% of time	32 ± 8% of cases
Caries	% of time	Duration	Caries	%(± se) of time	Duration (± se)
			
(a) filling			(a) all caries		
Moderate pain	20% of time	2 months	Moderate pain	34 ± 5% of time	^(1)^29 ± 9 months
Moderate anxiety	20% of time	2 months	Moderate anxiety	34 ± 10% of time	^(1)^13 ± 5 months
(b) extraction			(b) all caries		
Extreme pain	20% of time	2 weeks	Extreme pain	59 ± 14% of time	^(2)^50 ± 23 weeks
Moderate anxiety	20% of time	2 weeks	Moderate anxiety	34 ± 10% of time	^(1)^51 ± 18 weeks

(a): Estimates are reported specific to level of problem (eg, moderate), dimension (eg, pain) and condition (eg, caries)

(1): Relative standard error = 30–39%

(2): Relative standard error = 40–49%

For comparison purposes the disability weights for oral conditions are presented in Table 8 along with a range of weights for other health conditions from the Australian Burden of Disease and Injury Study [7] classified into disability classes [17]. Some oral conditions such as dental aesthetics have very low weights, (e.g., 0.002). Conditions such as tooth wear and tooth fracture had weights comparable with moderate anaemia. Denture problems, failed restorations and periodontal disease were lower but comparable with the weight for mild asthma. Dentinal sensitivity and caries were comparable with the weight for an episode of influenza. Pulpal infection, which had the highest weight of all oral conditions, had a weight comparable with acute sinusitis and lower than other conditions such as severe anaemia and gastroenteritis.

**Table 8:** Comparison of disability weights for a range of health conditions by source.

Disability class	Disability weights	Health condition	Disability Weight	Source	Oral/dental conditions
1	0.00–0.01	Aesthetics (dental)	0.002	Current study	Yes
		Anaemia (mild)	0.005	ABDS	
2	0.01–0.05	Wear (tooth)	0.011	Current study	Yes
		Anaemia (moderate)	0.011	ABDS	
		Fracture (tooth)	0.014	Current study	Yes
		Failed restoration	0.019	Current study	Yes
		Periodontal disease	0.023	Current study	Yes
		Denture problem	0.026	Current study	Yes
		Asthma (mild)	0.030	ABDS	
		Sensitivity (dentinal)	0.040	Current study	Yes
		Caries	0.044	Current study	Yes
		Influenza (episode)	0.047	ABDS	
3	0.05–0.10	Chronic back pain (episode)	0.060	ABDS	
		Sinusitis (acute)	0.061	ABDS	
		Pulpal infection	0.069	Current study	Yes
		Anaemia (severe)	0.090	ABDS	
		Gastroenteritis	0.093	ABDS	
4	0.10–0.15	Mild depression (episode)	0.140	ABDS	
5	0.15–0.20	Measles	0.152	ABDS	
		Trachoma (moderate)	0.170	ABDS	
		Conjunctivitis	0.180	ABDS	
6	0.20–0.30	Asthma (severe)	0.230	ABDS	
		Tuberculosis	0.295	ABDS	
7	0.30–0.40	Moderate depression (episode)	0.350	ABDS	
8	0.40–0.50	Trachoma (severe)	0.430	ABDS	
9	0.50–0.65	Tetanus	0.612	ABDS	
10	0.65–0.80	Severe depression (episode)	0.760	ABDS	
11	0.80–1.00	Cancer (terminal stage)	0.930	ABDS	

ABDS: Australian Burden of Disease and Injury Study

## Discussion

### Response

Response rates to the survey were adequate for both the dentist and patient questionnaires [18]. Comparison of respondents against estimates for private general practice and the Australian population indicated a slightly higher percentage of female patients compared to the population consistent with higher reported visiting rates by females [16], but both place of birth and time since last visit was similar. While dental insurance was higher, the percentage of check-up visits was lower among patients indicating a higher percentage of dental problems for patients compared to the population. The method of sampling patients showed that response rates tended to be higher among dentists who had to sample fewer patients consistent with a lower response burden, but selection of an optimal collection methodology requires consideration of efficiency of collection as well as response rates.

### Burden of disease approach

The burden of disease approach is grounded on the use of the DALY to quantify the burden of disease that treats 'like as like' within an information set of health conditions of individuals [19]. While use of DALYs has been criticised on the basis of its assumptions and value judgements [20], Murray & Acharya [19] argue that the widespread use of DALYs makes them a convenient tool for comparative burden of disease and cost-effectiveness analyses.

The EuroQol was developed as a standardised non-disease-specific instrument for describing and valuing health-related quality of life [12] and hence represents the best method to quantify DALYs. The EuroQol is intended to complement other forms of quality of life measures and it was purposefully developed to generate a generic index of health. Any classified health state can be valued using preferences elicited from a general population [12], and values can be modelled from such data sets [13,21]. The EuroQol is widely used internationally and reported to have adequate construct and convergent validity, but is highly skewed and has relatively poor sensitivity especially in relation to disease-based outcomes research [22].

The six dimensions of the EuroQol were used as a standardized description of health status in the development of disability weights for the Dutch Disability Weights Study [17]. The Australian Burden of Disease and Injury Study adopted the Dutch weights where possible. While both DALYs and the EuroQol instrument have their critics, if these approaches continue to influence policy decisions as to the scope and importance of oral disease then there will be an increasing need to assess the validity of the estimates and to address any shortcomings that are identified.

### Assumptions of disability weights

Differences in disability weights between the Australian Burden of Disease and Injury Study and this paper probably reflect a lack of quantitative data in the Dutch study related to the nature of symptoms experienced by persons with dental conditions. The data from this paper shows that many common dental conditions are associated with symptoms that on average were more severe and of longer duration than previously assumed by Stouthard et al. [17].

The calculation of disability weights in this paper was based on the use of EuroQol health state descriptions as in the Dutch study, but instead of using a panel approach to elicit valuations we adopted a model-based approach to estimate health state valuations for each individual response and then derive a disability weight as the average of those individual estimates. Such a model-based approach was also used as a source of validation in the original Dutch study but was not developed in detail due to the lack of an adequate statistical model at the time of development [23]. Two strategies are recognised as ways of arriving at a link between epidemiological data and disability weights [24]. The first one is derivation of disease-specific disability weights using health state descriptions with a disease label. The second approach, adopted in this study, is derivation of disability weights using generic descriptions of health states associated with specific diseases. In this study disability associated with oral disease was described using a generic measure (the EuroQol) valued by applying an existing formula [13]. The advantage of this approach is the transparency of the valuation task and the use of the formula provides the facility to cover generic health states without additional valuation studies [24].

Disability weights reflect health state valuations whereby weights are assigned to health states that are worse than ideal health. A range of methods can be used to elicit health state valuations including visual analogue scale, time trade-off and person trade-off. The visual analogue scale method uses a scale anchored by the best imaginable health state at 100 and death at 0, with respondents asked to indicate the exact point on the scale they would place a particular health state relative to the best imaginable health, death and all other health states. Time trade-off methods ask respondents to imagine choosing between the two options of remaining in a particular health state for 10 remaining years of life or be restored to perfect health but live for a shorter time. Person trade-off methods ask respondents to choose between two different programmes, one that would prevent the deaths of 100 perfectly healthy individuals and one that would prevent the onset of a particular health problem in a certain number of healthy people. While there is little agreement as to which method is most appropriate [25], it has been shown that visual analogue scale methods tend to give lower values for particular health states, than time trade-off methods, which give lower health state values than person trade-off methods. The additive model weights in this study were derived from a U.K. study based on valuations produced from visual analogue scale and time trade-off methods [13] whereas the multiplicative model weights were derived from a Dutch study based on visual analogue scale and person trade-off methods [23]. It could be argued that since the Australian Burden of Disease and Injury Study used disability weights based on person trade-off methods and this study used disability weights based on time trade-off methods that any differences between the disability weights from this study with the Australian Burden of Disease and Injury Study could reflect differences in methodology. Also, the Australian Burden of Disease and Injury Study used a multiplicative model fitted to the Dutch weights for 153 disease sequelae or stages as multiplicative multi-attribute functions were preferred for providing better fit to observed preference data than additive models [7]. However, the final adjusted disability weights derived from the additive model produced results that were consistent with, but slightly lower than, the multiplicative model. Therefore methodological issues stemming from valuation and modelling strategies do not seem to explain the differences that were observed.

One consideration arising from the disability weights derived in the present study was the reliance on the use of data from patients seeking care. The experience of dental problems from the perspective of a patient may be different than that from the population as a whole. If the symptoms experienced by patients were more severe compared to the general population then some further adjustment may be required to reduce the disability weights appropriately. However, it would not be right to assume that most patients attending for dental care would be symptomatic as patient-based data have shown that the majority of patients reported no problems on the six EuroQol dimensions (ranging from 69.7% for pain/discomfort to 98.6% for self-care), with 39.6% of patients reporting symptoms on one or more of the six dimensions [26]. Conversely, patients who are unable or unwilling to seek care can be expected to have a longer duration, and perhaps severity of dental symptoms and associated health problems than subjects in this study. There is some evidence of a symptom iceberg with respect to oral and facial pain, with Canadian population data showing that less than one in two who experience such pain consult a dentist or physician [27]. Since there are plausible arguments as to why patient-based estimates might reflect either more severe or less severe conditions the question of possible bias in a patient population remains open and perhaps could be settled by further research. An important design feature of this study was the use of dentists to diagnose the oral health conditions that were subsequently reported on by the patients. Further refinement of these disability weights could be achieved through the use of an oral health survey based on a population sample that also uses a linked questionnaire to survey the experience of oral health problems. It may also be the case that further refinements to the algorithm for estimating disability weights that incorporates the cognitive dimension may also increase the size of weights although oral health conditions related to this dimension were not as prevalent as other dimensions that were included such as pain/discomfort and anxiety/depression. The weights derived from the multiplicative model included the cognitive dimension and this may help explain why they were observed to be slightly larger than the weights derived from the additive model. Detailed prospective data would be required to evaluate whether persons report the experience of their symptoms accurately or are more influenced by the end-stage of their disease experience than by the average experience over the period of their symptoms. Generic measures such as SF-36 have been found to be less sensitive to changes in oral health and to exhibit limited construct validity in comparison to specific measures of oral health [28]. Despite being a generic measure the EuroQol has shown discriminant validity in relation to a range of dental patient, visit and oral health measures [29]. However, in general there can be problems assigning disability weights to diseases with high prevalence and low severity, relating to the lack of differentiation at this low end of the scale [24].

### Implications of oral health disability weights

The findings from this study indicate that oral health conditions may account for a considerably higher level of DALYs than previously thought, due to the lack of quantitative data on the nature of dental conditions. While Australia has not had another national oral health survey since the initial survey of 1987–88, there have been other studies that suggest that dental problems are common [2,4], and account for large amounts of health care costs [3]. Further work could be done to incorporate the revised disability weights for oral health into new estimates of the burden of disease in order to estimate the impact that such revisions to the disability weights have on the number of DALYs, and how this affects the ranking of oral health problems in relation to other health conditions.

## Conclusions

Compared to the Australian Burden of Disease and Injury Study the adjusted disability weights for oral health conditions in this study were higher for comparable oral conditions of caries (0.044 versus 0.005 for caries involving a filling and 0.014 for caries involving an extraction), periodontal disease (0.023 versus 0.007) and denture problems (0.026 versus 0.004 for edentulism). In addition there were a range of common oral health problems such as pulpal infection, failed restorations and tooth fracture that were not included in the Australian Burden of Disease and Injury Study which had relatively high disability weights. The inclusion of a fuller range of oral health conditions along with revised disability weights would result in oral health accounting for a much larger amount of disability than originally estimated.

## Competing interests

None declared.

## Authors' contributions

DSB and AJS were chief investigators on the grants obtained to fund the study. DSB performed data collection, analysis and drafting of the manuscript. AJS participated in the design and coordination of the study, and completion of the manuscript. All authors read and approved the manuscript.

## Supplementary Material

Additional File 1 Appendix 1: algorithm used to calculate disability weights from the additive model. Appendix 2: algorithm used to calculate disability weights from the multiplicative model Click here for file

## References

[B1] Australian Institute of Health and Welfare (2001). Health expenditure bulletin no 17: Australia's health services expenditure to 1999–00.

[B2] Spencer AJ, Lewis JM (1988). The delivery of dental services: information, issues and directions. Community Health Studies.

[B3] Crowley S, Antioch K, Carter R, Waters AM, Conway L, Mathers C (1992). The cost of diet-related disease in Australia.

[B4] Australian Institute of Health and Welfare (2000). Australia's health The seventh biennial report of the Australian Institute of Health and Welfare.

[B5] Murray CJL (1994). Quantifying the burden of disease: the technical basis for disability-adjusted life years. Bulletin of the World Health Organization.

[B6] Murray CJL, Lopez AD (1994). Quantifying disability: data, methods and results. Bulletin of the World Health Organization.

[B7] Mathers C, Vos T, Stevenson C (1999). The burden of disease and injury in Australia.

[B8] Barnard PD (1993). National oral health survey of Australia, 1987–88.

[B9] AIHW Dental Statistics and Research Unit (1998). Australia's oral health and dental services Adelaide: The University of Adelaide.

[B10] Dillman DA (1978). Mail and telephone surveys The total design method.

[B11] Brennan DS, Spencer AJ (2002). Dentists' practice activity in Australia: 1983–84 to 1998–99.

[B12] Brooks R (1996). EuroQol: the current state of play. Health Policy.

[B13] Dolan P (1997). Modeling valuations for EuroQol health states. Medical Care.

[B14] StataCorp (1999). Stata statistical software: release 60 College Station, TX: Stata Corporation.

[B15] Australian Bureau of Statistics (2002). Australian demographic statistics – March quarter 2002.

[B16] Carter KD, Stewart JF, Spencer AJ (2001). National dental telephone interview survey 1999.

[B17] Stouthard MEA, Essink-Bot M-L, Bonsel GJ (2000). Disability weights for diseases. A modified protocol and results for a Western European region. European Journal of Public Health.

[B18] Mangione TW (1995). Mail surveys Improving the quality.

[B19] Murray CJL, Acharya AK (1997). Understanding DALYs. Journal of Health Economics.

[B20] Anand S, Hanson K (1997). Disability-adjusted life years: a critical review. Journal of Health Economics.

[B21] Dolan P, Roberts J (2002). Modelling valuations for EQ-5D health states. An alternative model using differences in valuations. Med Care.

[B22] Bowling A (2001). Measuring disease A review of disease-specific quality of life measurement scales.

[B23] Stouthard MEA, Essink-Bot M-L, Bonsel GJ, Barendregt JJ, Kramers PGN, van de Water HPA, Gunning-Schepers LJ, van der Maas PJ (1997). Disability weights for diseases in the Netherlands.

[B24] Essink-Bot M-L, Bonsel GJ, Murray CJL, Saloman JA, Mathers CD, Lopez AD (2002). How to derive disability weights. In Summary Measures of Population Health Concepts, Ethics, Measurementand Applications.

[B25] Saloman JA, Murray CJL, Murray CJL, Saloman JA, Mathers CD, Lopez AD (2002). Estimating health state valuations using a multiple-method protocol. In Summary Measures of Population Health Concepts, Ethics, Measurement and Applications.

[B26] Brennan DS, Spencer AJ (2004). Dimensions of oral health related quality of life measured by the EQ-5D+ and OHIP-14. Health Qual Life Outcomes.

[B27] Locker D (1988). The symptom iceberg in dentistry. Treatment-seeking in relation to oral and facial pain. J Can Dent Assoc.

[B28] Allen PF, McMillan AS, Walshaw D, Locker D (1999). A comparison of the validity of generic-and disease-specific measures in the assessment of oral health-related quality of life. Community Dent Oral Epidemiol.

[B29] Brennan DS, Spencer AJ Comparison of a generic and a specific measure of oral health related quality of life. Community Dent Health.

