# Supplementary material for: Disability weights for the burden of oral disease in South Australia
Source: Popul Health Metr. 2004 Sep 3;2:7. doi: 10.1186/1478-7954-2-7 (PMC517729; doi:10.1186/1478-7954-2-7)
Supplement: Additional File 1 — Appendix 1: algorithm used to calculate disability weights from the additive model. Appendix 2: algorithm used to calculate disability weights from the multiplicative model [file 1478-7954-2-7-S1.pdf]

Appendix 1: algorithm used to calculate disability weights from the additive model

The algorithm used for the unadjusted disability weight (DW_a) was as follows:

$$DW_a = 0.081 + (0.069\times MO) + (0.104\times SC) + (0.036\times UA) + (0.123\times PD) + (0.071\times AD) + (0.176\times M2) + (0.006\times S2) + (0.022\times U2) + (0.140\times P2) + (0.094\times A2) + (0.269\times N3)$$

The algorithm for the disability weight adjusted by percentage of time affected (DW_b) was as follows:

$$DW_b = 0.081 + (0.069\times MO\times P_1) + (0.104\times SC\times P_2) + (0.036\times UA\times P_3) + (0.123\times PD\times P_4) + (0.071\times AD\times P_5) + (0.176\times M2\times P_1) + (0.006\times S2\times P_2) + (0.022\times U2\times P_3) + (0.140\times P2\times P_4) + (0.094\times A2\times P_5) + (0.269\times N3)$$

where P_1 to P_5 is percentage of time experiencing symptoms in relation to that dimension

The algorithm for the disability weight adjusted by the constant (DW_c) was as follows:

$$DW_c = DW_b - 0.081$$

Coding of variables in disability weight algorithms – additive model

**Variable**	**Definition**
Constant	Constant (=0.081): associated with any move away from full health
MO	MO=1 if mobility is level 2 (some/moderate problem);<br>MO=2 if mobility is level 3 (extreme problem);<br>MO=0 otherwise (no problem)
SC	SC=1 if self-care is level 2 (some/moderate problem);<br>SC=2 if self-care is level 3 (extreme problem);<br>SC=0 otherwise (no problem)
UA	UA=1 if usual activities is level 2 (some/moderate problem);<br>UA=2 if usual activities is level 3 (extreme problem);<br>UA=0 otherwise (no problem)
PD	PD=1 if pain/discomfort is level 2 (some/moderate problem);<br>PD=2 if pain/discomfort is level 3 (extreme problem);<br>PD=0 otherwise (no problem)
AD	AD=1 if anxiety/depression is level 2 (some/moderate problem);<br>AD=2 if anxiety/depression is level 3 (extreme problem);<br>AD=0 otherwise (no problem)
M2	M2=1 if mobility is level 3 (extreme problem);<br>M2=0 otherwise
S2	S2=1 if self-care is level 3 (extreme problem);<br>S2=0 otherwise
U2	U2=1 if usual activities is level 3 (extreme problem);<br>U2=0 otherwise
P2	P2=1 if pain/discomfort is level 3 (extreme problem);<br>P2=0 otherwise
A2	A2=1 if anxiety/depression is level 3 (extreme problem);<br>A2=0 otherwise
N3	N3=1 if any dimension is level 3 (extreme problem);<br>else N3=0

Appendix 2: algorithm used to calculate disability weights from the multiplicative model

The algorithm used for the unadjusted disability weight (DW_d) was as follows:

$$\log(w) = 0 + (-0.247 \times d_{12}) + (-0.5419 \times d_{13}) + (-0.12455 \times d_{22}) + (-0.5718 \times d_{23}) + (0.000 \times d_{32}) + (-0.2051 \times d_{33}) + (-0.0576 \times d_{42}) + (-0.5039 \times d_{43}) + (-0.2284 \times d_{52}) + (-0.742 \times d_{53}) + (-0.1915 \times d_{62}) + (-0.5636 \times d_{63}) + (-0.01 \times s) + (-0.1228 \times p)$$

$$DW_d = 1 - e^{\log(w)}$$

The algorithm for the disability weight adjusted by percentage of time affected (DW_e) was as follows:

$$\log(w) = 0 + (-0.247 \times d_{12} \times P_1) + (-0.5419 \times d_{13} \times P_1) + (-0.12455 \times d_{22} \times P_2) + (-0.5718 \times d_{23} \times P_2) + (0.000 \times d_{32} \times P_3) + (-0.2051 \times d_{33} \times P_3) + (-0.0576 \times d_{42} \times P_4) + (-0.5039 \times d_{43} \times P_4) + (-0.2284 \times d_{52} \times P_5) + (-0.742 \times d_{53} \times P_5) + (-0.1915 \times d_{62} \times P_6) + (-0.5636 \times d_{63} \times P_6) + (-0.01 \times s) + (-0.1228 \times p)$$

$$DW_e = 1 - e^{\log(w)}$$

where P_1 to P_6 is percentage of time experiencing symptoms in relation to that dimension and e is the base of the natural logarithm

Coding of variables in disability weight algorithms – multiplicative model

Variable	Definition
d_{12}	d_{12}=1 if mobility is level 2 (some/moderate problem);<br>d_{12}=0 otherwise
d_{13}	d_{13}=1 if mobility is level 3 (extreme problem);<br>d_{13}=0 otherwise
d_{22}	d_{22}=1 if self-care is level 2 (some/moderate problem);<br>d_{22}=0 otherwise
d_{23}	d_{23}=1 if self-care is level 3 (extreme problem);<br>d_{23}=0 otherwise
d_{32}	d_{32}=1 if usual activities is level 2 (some/moderate problem);<br>d_{32}=0 otherwise
d_{33}	d_{33}=1 if usual activities is level 3 (extreme problem);<br>d_{33}=0 otherwise
d_{42}	d_{42}=1 if pain/discomfort is level 2 (some/moderate problem);<br>d_{42}=0 otherwise
d_{43}	d_{43}=1 if pain/discomfort is level 3 (extreme problem);<br>d_{43}=0 otherwise
d_{52}	d_{52}=1 if anxiety/depression is level 2 (some/moderate problem);<br>d_{52}=0 otherwise
d_{53}	d_{53}=1 if anxiety/depression is level 3 (extreme problem);<br>d_{53}=0 otherwise
d_{62}	d_{62}=1 if cognition is level 2 (some/moderate problem);<br>d_{62}=0 otherwise
d_{63}	d_{63}=1 if cognition is level 3 (extreme problem);<br>d_{63}=0 otherwise
s	s=1 if all dimensions are level 1 (no problem) but disease present;<br>else s=0
p	p=1 if prognosis is uncertain;<br>else p=0
